# Computational Phenotypic Drug Discovery for Anticancer Chemotherapy: PTML Modeling of Multi-Cell Inhibitors of Colorectal Cancer Cell Lines

**DOI:** 10.3390/ijms262311453

**Published:** 2025-11-26

**Authors:** Alejandro Speck-Planche, M. Natália D. S. Cordeiro

**Affiliations:** LAQV/REQUIMTE, Department of Chemistry and Biochemistry, Faculty of Sciences, University of Porto, 4169-007 Porto, Portugal

**Keywords:** PTML, topological indices, multilayer perceptron, fragment, fragment-based topological design, colorectal cancer

## Abstract

Colorectal cancer is one of the most dangerous neoplastic diseases in terms of both mortality and incidence. Thus, anti-colorectal cancer agents are urgently needed. Computational approaches have great potential to accelerate the phenotypic discovery of versatile anticancer agents. Here, by combining perturbation-theory machine learning (PTML) modeling with the fragment-based topological design (FBTD) approach, we provide key computational evidence on the computer-aided de novo design and prediction of new molecules virtually exhibiting multi-cell inhibitory activity against different colorectal cancer cell lines. The PTML model created in this study achieved sensitivity and specificity values exceeding 80% in training and test sets. The FBTD approach was employed to physicochemically and structurally interpret the PTML model. These interpretations enabled the rational design of six new drug-like molecules, which were predicted as active against multiple colorectal cancer cell lines by both our PTML model and a CLC-Pred 2.0 webserver, with the latter being a well-established virtual screening tool for early anticancer discovery. This work confirms the potential of the joint use of PTML and FBTD as a unified computational methodology for early phenotypic anticancer drug discovery.

## 1. Introduction

Colorectal cancer (CRC) stands as a significant global health challenge, ranking among the most prevalent neoplastic diseases and being a leading cause of cancer-related mortality worldwide [1]. In developed countries such as the US, more than 150,000 new CRC cases and over 50,000 deaths are expected to occur this year, 2025 [2]. It is widely recognized that CRC involves an intricate combination of genetic mutations, epigenetic alterations, and environmental factors, leading to uncontrolled proliferation of colonic epithelial cells and metastasis [3]. Despite advances in screening and therapeutic solutions, including chemotherapy, immunotherapy, and targeted therapies, CRC prognosis remains poor in advanced stages due to tumor heterogeneity and drug resistance [4].

These challenges indicate the urgent need for the discovery of new anticancer agents capable of targeting CRC proliferation.

Although experimental screening remains the gold standard in early anticancer drug discovery for evaluating the efficacy of anticancer compounds at both target-based and phenotypic levels [5], these approaches are resource-intensive and time-consuming. In this context, in silico approaches, widely regarded as powerful tools in modern drug discovery campaigns, have played an important role in rationalizing the discovery of anti-CRC agents. In silico approaches such as pharmacophore modeling [6], network-based analysis [7,8], molecular docking and molecular dynamics simulations [6,7,8,9,10,11,12,13], quantum mechanical calculations [10,13], and machine learning [7,13] have proven highly useful. However, several limitations persist, including (a) the reliance on small chemical datasets (limiting coverage of chemical space), (b) prediction of activity against only one cancer-related target (e.g., protein, or cancer cell line), which reduces the potential pharmacological applicability of the modeled/predicted chemicals, and (c) insufficient interpretability of physicochemical properties and structural features (hindering the rational design of multi-target ligands or multi-cell inhibitors with anticancer versatility.

Advanced models based on perturbation-theory machine learning (PTML) have demonstrated the potential to overcome these limitations [14,15]. In this sense, PTML models have been successfully applied in diverse pharmacology-related areas, including antimicrobial research [16,17,18,19,20,21,22], neurodegenerative diseases [23,24,25,26,27], immunology [28,29], nanomedicine [30,31,32,33,34,35], and antineoplastic drug discovery [36,37,38]. Moreover, PTML models can be coupled with the fragment-based topological design (FBTD) approach to enable the de novo design of molecules predicted to possess specific bioactivity profiles [16,36,39].

To date, there are no reports employing PTML modeling for the rational in silico design of CRC-targeted agents capable of simultaneously considering phenotypic aspects affecting cancer proliferation (e.g., cell doubling times and microsatellite characteristics). In this study, we provide key computational evidence that demonstrates that a computational framework, combining a PTML model based on a multilayer perceptron network (PTML-MLP) with the FBTD, can facilitate both the prediction and the de novo design of drug-like molecules with multi-cell inhibitory activity against different CRC cell lines. The study is fully computational, and the predictions and designed molecules generated by the PTML–FBTD computational framework are intended to guide future experimental validation rather than constitute biological confirmation.

## 2. Results and Discussion

### 2.1. PTML Model: Performance

The PTML-MLP model presented in this study used as inputs the so-called multi-label topological indices *D*[*GTI*]*ej*, which fuse information on the chemical structure of molecules and a combination of experimental aspects (*ej*) associated with the CRC cell lines. In this context, each *ej* combines the doubling time of each CRC cell line (*dt*) [40], the specific type of CRC cell line (*ct*) [41], and the microsatellite characteristics (*mc*) of each CRC cell line [41,42]. The best PTML-MLP model has the notation MLP 20-50-2. This means that there were 20 input nodes (*D*[*GTI*]*ej* indices), 50 hidden neurons, and two output nodes; hyperbolic tangent was used as the activation function in both hidden and output layers. The aforementioned PTML-MLP model notation provides information about its topology, which, together with the number of training cases employed (*T* = 4108), led to the value of the parameter *ρ* = 3.57 (the formula for the calculation of *ρ* appears discussed in the Materials and Methods section). This *ρ* value indicates that the PTML-MLP model is not overfitting the data [43]. A summary of the different *D*[*GTI*]*ej* is provided in Table 1.

To analyze the performance of the PTML-MLP model, we used both global and local statistical metrics. We used sensitivity (*Sn*), specificity (*Sp*), and the normalized Matthew’s correlation coefficient (*nMCC*) [44] as the global metrics. From Table 2, one can see that a value of *Sn* = 89% was achieved in the training set, while in the test set, *Sn* > 83%. For *Sp*, values higher than 89% and 84% are reported for training and test sets, respectively.

All this means that the PTML-MLP model can correctly classify or predict chemicals as either active (*CC*_Active_) or inactive (*CC*_Inactive_), relative to the total number of chemicals labeled as active or inactive (*N*_Active_ and *N*_Inactive_, respectively). Furthermore, for the metric *nMCC,* the values are close to 1, which means that the correlation between the observed [*ACRC*(*ej*)] and the predicted [*PACRC*(*ej*)] categorical values of anti-colorectal cancer activity is very strong. For the dataset used in the present study, chemical and biological data can be found in Supplementary Materials S1. Furthermore, details on the prediction outcome for each chemical in the dataset used to develop and validate the PTML-MLP model are available in Supplementary Materials S2. It is important to note that chemicals/cases annotated as active have GI_50_ ≤ 1900 nM (GI_50_ is the concentration causing 50% growth inhibition), while the remaining cases are annotated as inactive.

We also employed local statistical metrics known as local sensitivities [*Sn*(*dt*), *Sn*(*ct*), and *Sn*(*mc*)] and specificities [*Sp*(*dt*), *Sp*(*ct*), and *Sp*(*mc*)] as they allow the assessment of the PTML-MLP model’s performance by considering the elements/aspects of *ej*. Regarding the biological aspect *dt*, both *Sn*(*dt*) and *Sp*(*dt*) presented values in the interval 87–91.1% in the training set; in the test set, for these local metrics, the achieved interval was 80–86.3%. For the element *ct*, *Sn*(*ct*) and *Sp*(*ct*) displayed values in the range 84–93.2% in the training set, while also exhibiting a range of 79–87.1% in the test set. For the biological aspect *mc*, the values of the local metrics were *Sn*(*mc*) > 88% and *Sp*(*mc*) > 87% in the training set, while for the test set, *Sn*(*mc*) > 82% and *Sp*(*mc*) > 83% were obtained. Altogether, both global and local statistical metrics demonstrate that the PTML-MLP can accurately predict the anti-CRC activity. The PTML-MLP model does so by simultaneously and explicitly considering the growth/proliferation of the CRC cell lines (doubling times—*dt*), the specific types of CRC cell lines (*ct*), and the microsatellite characteristics (*mc*). Specific values of the local metrics associated with each label can be found in Supplementary Materials S2.

**Figure 1:**
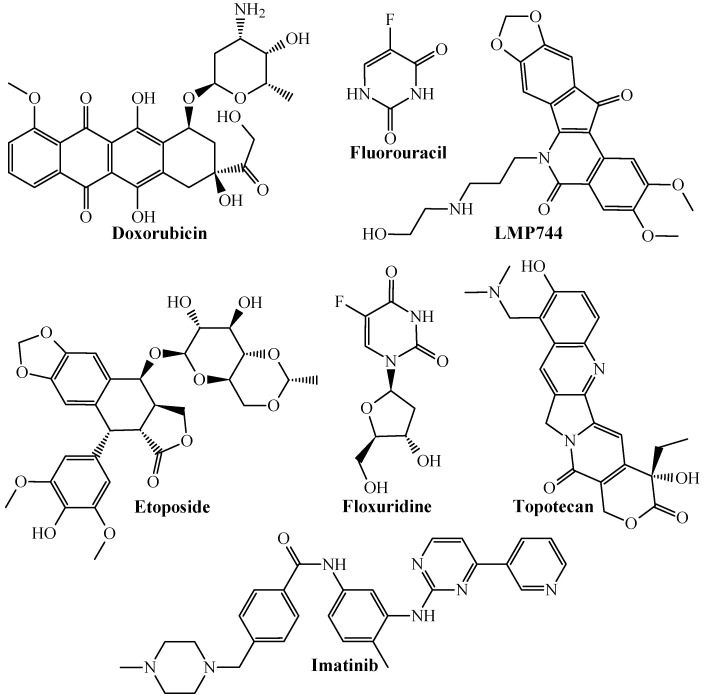
Chemical structures of anticancer drugs correctly predicted by the PTML-MLP model.

At a deeper chemical level, Figure 1 illustrates that the PTML-MLP model can correctly predict/classify the anti-CRC activity of well-established anticancer drugs.

**Figure 2:**
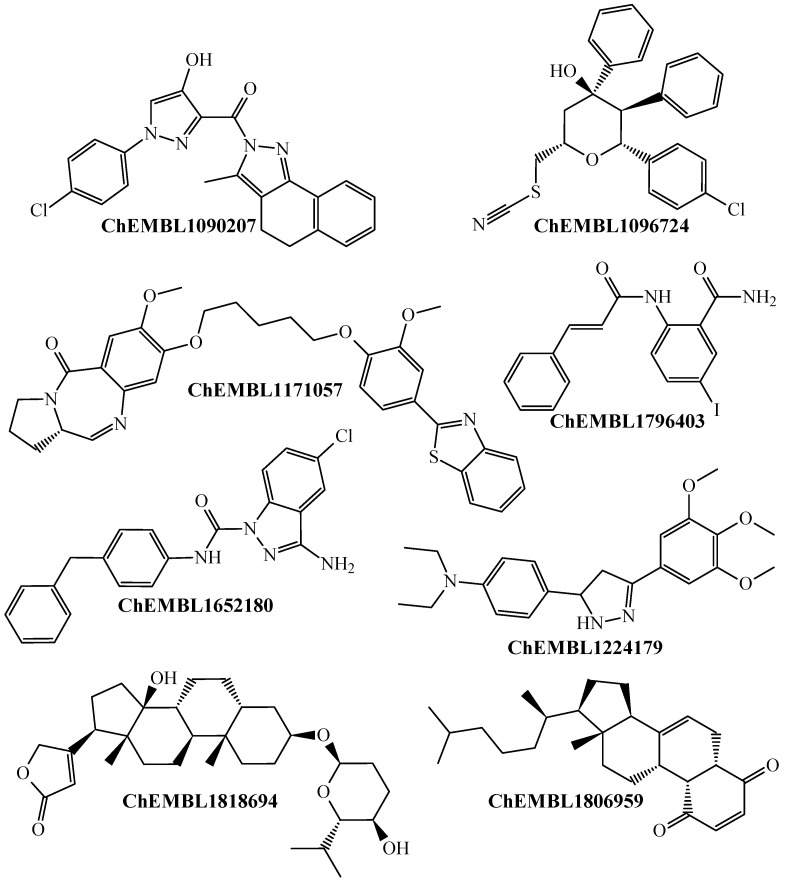
Chemicals labeled and correctly predicted by the PTML-MLP model as multi-cell inhibitors against the CRC cell lines.

The anticancer drugs (both investigational and approved by the Food and Drug Administration—FDA) with in vitro inhibitory activity against one or more CRC cell lines, which were correctly predicted by the PTML-MLP model are those annotated as ChEMBL53463 (doxorubicin), ChEMBL185 (fluorouracil), ChEMBL126159 (LMP744), ChEMBL44657 (etoposide), ChEMBL917 (floxuridine), ChEMBL84 (topotecan), and ChEMBL941 (imatinib). This indicates that our PTML-MLP model can identify chemical patterns associated with drugs with well-established anti-CRC activity at the in vitro level. Our PTML-MLP model also excels in identifying/predicting new chemical patterns (Figure 2), which are different from those present in anticancer drugs.

For simplicity, Figure 2 illustrates a non-exhaustive list of molecules that can be found in the dataset of the present study (Supplementary Materials S1 and S2). This means that the molecules in the aforementioned figure have been experimentally tested; in particular, they have been assayed against all the combinations of *ej* depicted in Table 3, being reported as multi-cell inhibitors of all seven CRC cell lines. Despite belonging to diverse chemical scaffolds, our PTML-MLP model could correctly predict the multi-cell anti-CRC activity of all the molecules in Figure 2 (and many others reported in Supplementary Materials S1 and S2). These results demonstrate that our PTML-MLP model can serve as a computational tool for predicting or discovering novel chemicals (in virtual screening scenarios) with versatile anti-CRC activity.

Finally, we determined the applicability domain (AD) of the PTML-MLP model using a variation in the bounding box (descriptor space) as reported in recent studies [16,36,45]. In doing so, for each of the 20 *D*[*GTI*]*ej* indices present in the PTML-MLP model, we calculated a local score denoted as LSAD_*D*[*GTI*]*ej*. For a query chemical and a given *D*[*GTI*]*ej* index, LSAD_*D*[*GTI*]*ej* was a categorical variable, which took the value of 1 if the *D*[*GTI*]*ej* value of that chemical fell between the minimum and maximum *D*[*GTI*]*ej* values (with the minimum and maximum values being calculated from chemicals in the training set that were correctly classified by the PTML-MLP model); otherwise, the LSAD_*D*[*GTI*]*ej* took the value of zero. This operation was repeated for all the chemicals in the dataset and the 20 *D*[*GTI*]*ej* indices in the PTML-MLP model. As a result, is chemical was associated with LSAD_*D*[*GTI*]*ej* values. Next, the total applicability domain score (TSAD) was calculated for each chemical. If TSAD = 20, the chemical was considered inside the AD; otherwise, it was deemed outside the AD and regarded as an unreliable prediction. In the end, 5475 out of 5478 chemicals/cases were inside the AD of the PTML-MLP model (Supplementary Materials S2).

### 2.2. Physicochemical Interpretation of the PTML-MLP Through the FBTD Approach

The FBTD approach is suitable to perform physicochemical and structural interpretation of non-linear machine learning models, and, in particular, those based on the PTML philosophy [16,36,45]. In this sense, in the context of PTML modeling, and applied to the present study, FBTD comprises three steps, namely the estimation of the relative influence of the *D*[*GTI*]*ej* indices in our PTML-MLP model, the tendency of variation in the *D*[*GTI*]*ej* indices, and the gathering of information regarding different subgraphs and their corresponding molecular fragments, which may be responsible for the enhancement of the anti-CRC activity against the different CRC cell lines.

**Figure 3:**
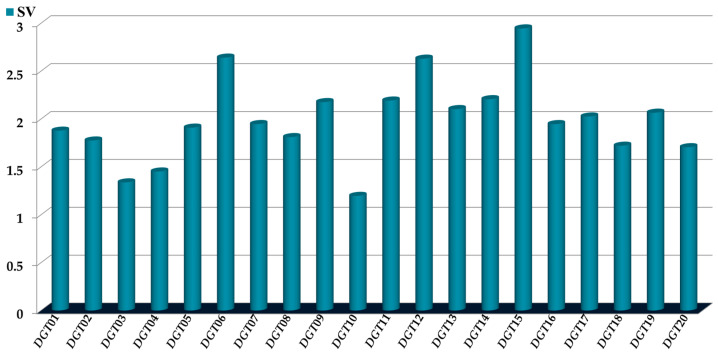
Relative importance of the *D*[*GTI*]*ej* indices in the PTML-MLP model, assessed through their *SVs*.

For the first step, we computed the sensitivity values (*SVs*) of the *D*[*GTI*]*ej* indices, which were used as inputs to build the PTML-MLP model (Figure 3). By definition, *SVs* are quantitative measures of the importance/discriminative power of inputs in a machine learning model [46].

Therefore, the *D*[*GTI*]*ej* indices with the highest *SVs* are not only the ones with the greatest influence/discriminatory power in the PTML-MLP model; they are also the ones containing the most important structural features and physicochemical properties that are desirable for enhancing the multi-cell anti-CRC activity. The second step involves assessing the tendency of variation in the *D*[*GTI*]*ej* indices (Table 4), meaning that the core idea is to determine whether the value of each *D*[*GTI*]*ej* index should increase or decrease to enhance the multi-cell anti-CRC activity.

Notice that Table 4 depicts two sets of averages, one associated with chemicals labeled and correctly classified as active, and the other reflecting the chemicals annotated and correctly classified as inactive. These two aforementioned averages are calculated from the training set. Now let us take, for example, the *D*[*GTI*]*ej* index *DGT*01; because the active-based average is smaller than the inactive-based average, this means that to increase the multi-cell CRC activity, the value of *DGT*01 is likely to decrease. When applying the same reasoning to the *D*[*GTI*]*ej* index *DGT*07, the active-based average is larger than the inactive-based average. This indicates that, to enhance the multi-cell CRC activity, the value of *DGT*07 is likely to increase.

**Figure 4:**
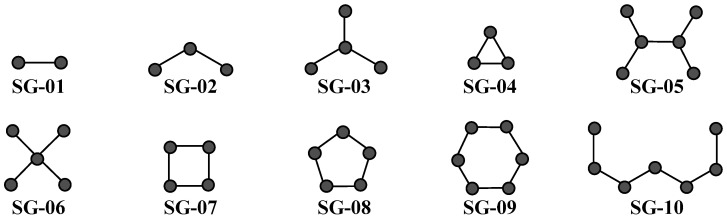
Most common subgraphs (*SGs*) described by the *D*[*GTI*]*ej* indices that characterize molecular fragments in the dataset used to create the PTML-MLP model.

The third step is related to the structural aspects associated with the information contained within the *D*[*GTI*]*ej* indices. In this sense, we provide subgraphs (Figure 4), i.e., generic structural representations from which molecular fragments (e.g., polar functional groups, aromatic portions, aliphatic chains and rings, and ramifications) can be easily analyzed. Thus, we will now proceed with the physicochemical and structural interpretations of the *D*[*GTI*]*ej* indices in the PTML-MLP model.

There are 10 *D*[*GTI*]*ej* indices (from *DGT*01 to *DGT*03, *DGT*05, from *DGT*07 to *DGT*10, *DGT*16, and *DGT*17) derived from the topological indices known as bond-based spectral moments. By definition, the bond-based spectral moments encode different physicochemical properties and can be expressed as a linear combination of the number of times in which fragments of different sizes appear in a molecule [47,48,49,50,51,52]. They can also describe 3D parameters such as dihedral angles [53]. This information remains in the *D*[*GTI*]*ej* indices derived from the topological indices known as bond-based spectral moments. The first of the *D*[*GTI*]*ej* indices, *DGT*01, indicates the diminution of the polarity-based property known as dipole moment, particularly in fragments containing the subgraphs SG-03 (favorable for isopropyl and tert-butyl groups, and to a lesser degree, a fluorine or chlorine attached to an aromatic carbon), SG-04 (e.g., presence of tert-butyl or tert-butoxy), and SG-06 (cyclopropane moiety); *DGT*01 is the thirteenth most important *D*[*GTI*]*ej* index in the PTML-MLP model. Following with polarity-based properties, we have *DGT*02, *DGT*05, and *DGT*08; they rank fifteenth, twelfth, and fourteenth, respectively. These *D*[*GTI*]*ej* indices describe the decrease in the polar surface area; while *DGT*02 and *DGT*05 take into account the global polar surface area, i.e., SG-01 subgraphs (with *DGT*02 being size-independent), *DGT*08 focuses on the same *SGs* as *DGT*01 plus the SG-07 subgraphs (cyclobutene preferred over its heteroatoms-containing counterparts), those prioritizing the same molecular fragments/functional groups. Notice that this does not mean that polar groups cannot be present; the key is that their presence should be reduced as much as possible, and therefore, if present, only one highly polar group (e.g., urea, amide, etc.) should be allowed.

At the same time, polarizability is also a very important physicochemical property. In this sense, the three *D*[*GTI*]*ej* indices derived from bond-based spectral moments, namely *DGT*03, *DGT*09, and *DGT*17 (ranking nineteenth, sixth, and ninth, respectively, among the most important *D*[*GTI*]*ej* indices in the PTML-MLP model), describe the diminution of the aforementioned property to increase the multi-cell anti-CRC activity. More specifically, *DGT*03 describes fragments involving the SG-03, SG-04, SG-06, and SG-07 subgraphs (four-membered rings), while *DGT*09 focuses only on SG-03 and SG-04; *DGT*17 is a measure of the global polarizability of a molecule (SG-01 subgraphs). Altogether, they indicate that the functional groups tert-butyl, trifluoromethyl, and tert-butoxy, trifluoromethoxy are very suitable, particularly when attached to rings. At the same time, high-polarizability atoms such as halogens other than fluorine, sulfur, and phosphorus should be avoided; if present in a molecule, only one of these atoms is allowed. Because aromatic rings are very important in most chemical structures with biological activity, their relatively high polarizability is detrimental (they contribute to the unfavorable increase in the global polarizability), and, to manage that, aliphatic portions (including aliphatic rings) should be introduced to separate any two aromatic systems. Oxygen-containing functional groups (in particular, methoxy, hydroxyl, and amide) are also very suitable for decreasing the polarizability.

Furthermore, hydrophobicity, atomic weight, and bond distance influence the multi-cell anti-CRC activity. On one side, the increase in the global hydrophobicity is characterized by *DGT*07 (SG-01 subgraphs), which prioritizes the presence of trihalomethyl groups, aromatic carbons (except those to which nitrogen or oxygen atoms are attached), tertiary amines, pyrrolic nitrogen atoms, as well as thiol and thioether groups and halogens (mainly Cl, Br, I) attached to rings. On the other hand, *DGT*10 involves the increase in the atomic weight in SG-03, SG-04, and SG-06 subgraphs, thus prioritizing the presence of trifluoromethyl and trifluoromethoxy, as well as the presence of Cl, Br, I, and S. In the case of *DGT*16, this is a *D*[*GTI*]*ej* index with steric effect implications because indicates the need to increase the bond distance (SG-01 subgraphs), making a molecule bigger; this can be achieved by introducing saturated aliphatic portions as well as Cl, Br, I, and S. It is important to highlight that *DGT*07, *DGT*10, and *DGT*16, are the tenth, twentieth, and eleventh most important *D*[*GTI*]*ej* indices in the PTML-MLP model.

In the PTML-MLP model reported in the present study, we have two *D*[*GTI*]*ej* indices derived from the topological descriptors known as atom-based connectivity indices [54,55,56,57,58,59,60]. These are measures of molecular accessibility, that is, the ability of different molecular regions/fragments to participate in both polar and non-polar interactions with their surrounding environment (solvent molecules, amino acids in the pocket of a protein, molecules present in different locations of a cell, etc.). The first of these *D*[*GTI*]*ej* indices is *DGT*11 (ranked as the fifth most influential), and the favorable diminution of its value is equivalent to increasing the number of heteroatoms in six-membered rings (SG-09), where aromatic rings are preferred over their aliphatic counterparts. The other *D*[*GTI*]*ej* index, *DGT*18 (ranked sixteenth), involves the decrease in the molecular accessibility in linear fragments formed by six bonds (SG-10 subgraphs—without counting bond order). The favorable diminution in the value of *DGT*18 indicates an augmentation in the number of heteroatoms present in both aliphatic portions and aromatic systems, as well as the increase in the number of ramifications and polysubstituted rings (including fused ring systems).

The remaining eight *D*[*GTI*]*ej* indices are derived from the topological descriptors named bond-based connectivity indices, which are direct measures of fragment-based contributions to the molecular volume [61,62,63,64,65]. In this context, *DGT*04 (ranked eighteenth) characterizes the diminution of the molecular volume in regions containing two-bond fragments (SG-02 subgraphs), indicating the need for ramifications in the central part of a molecule as well as polysubstituted and fused ring systems. The *D*[*GTI*]*ej* index *DGT*06 is the second most important in the PTML-MLP model and indicates the diminution of the number of fragments based on the SG-06 subgraphs; thus, having one group containing this fragment (such as tert-butyl, trifluoromethyl, and trifluoromethoxy) attached to a ring is allowed. On the other hand, *DGT*12 and *DGT*19 characterize the increase in the molecular volume of a molecule (i.e., SG-01 subgraphs, with *DGT*19 being size-independent). This means that most ramifications should appear in the periphery of a molecule; *DGT*12 and *DGT*19 rank third and eighth among the most important *D*[*GTI*]*ej* indices in the PTML-MLP model.

At the same time, *DGT*13 is the seventh most important *D*[*GTI*]*ej* index and describes the increase in the number of molecular fragments containing the SG-05 subgraphs. Examples of structural moieties containing these subgraphs are N,N-disubstituted amides, isopropyl, N,N-dimethylamino, tert-butyl, and trifluoromethyl groups, as well as fused ring systems. On the other hand, *DGT*14 (ranked fourth) implies the diminution of the number of five-membered rings (SG-08 subgraphs); if present, no more than two five-membered rings are allowed, and they should have substitutions in two or more positions.

The most important *D*[*GTI*]*ej* index in the PTML-MLP model is *DGT*15, which characterizes the diminution of the molecular volume in six-membered rings (SG-09), indicating the need for the presence of polysubstituted rings. Finally, *DGT*20 involves the increase in the molecular volume in six-bond fragments (SG-10 subgraphs); if ramifications are present, they should appear in the peripheral regions of a molecule.

### 2.3. Designing Novel Molecules Virtually Exhibiting Multi-Cell Anti-CRC Activity

**Figure 5:**
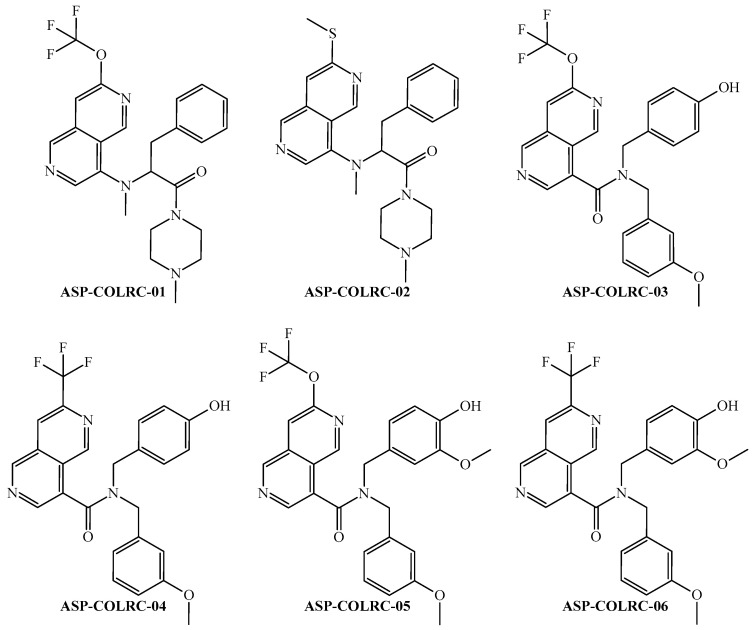
The FBTD approach applied to the design of new molecules virtually exhibiting multi-cell anti-CRC activity.

Using this joint interpretation, we designed six structurally related molecules (Figure 5) but with key chemical modifications to examine how the structural difference among molecules leads to marked outcomes in their predicted multi-cell anti-CRC activity.

The joint interpretation of all the *D*[*GTI*]*ej* indices in the PTML-MLP model suggests that aliphatic portions are very important in the chemical structure of a molecule expected to exhibit multi-cell anti-CRC activity, particularly if they appear in the central part of a molecule (separating the aromatic portions from each other) or heterocyclic rings; but two large aliphatic portions may be detrimental. Heteroaromatic fused systems are also very important, specifically if they are in the periphery of a molecule. The same goes for substituents such as tert-butyl, trifluoromethyl, and trifluoromethoxy, which can be attached to aromatic rings. The presence of a single highly polar functional group is important, and such a group can be in both the central and the peripheral parts of a molecule. Furthermore, low-polarizability groups such as hydroxyl, methoxy are very suitable as substituents in rings; the same goes for heavy atoms such as sulfur and halogens other than fluorine (the number of these heavy atoms should be as low as possible). Such structural features jointly discussed here are the ones present in the chemical structures of the designed molecules. To offer accurate computational/theoretical evidence regarding the potential of the designed molecules to exhibit multi-cell anti-CRC activity, the designed molecules were predicted by two tools (Table 5).

The results from Table 5 suggest that the six designed molecules exhibit predicted multi-cell anti-CRC activity because they were predicted by the PTML-MLP model as active (*ProbAct* > 50%) against at least 4 out of 7 combinations of *ej* (one per each CRC cell line), thus virtually exhibiting GI_50_ ≤ 1900 nM (the activity cutoff used when developing the PTML-MLP model). Particularly, the designed molecules ASP-COLRC-01 and ASP-COLRC-02 were predicted as active in 4 out of 7 combinations of *ej*, while ASP-COLRC-03 was predicted as active in 5 of the 7 aforementioned experimental aspects; the designed molecules ASP-COLRC-04, ASP-COLRC-05, and ASP-COLRC-06 were predicted to exhibit multi-cell anti-CRC activity by considering the seven combinations of *ej*. More details regarding the six designed molecules can be found in Supplementary Materials S3.

From a chemical point of view, there are marked structural differences among the designed molecules, which ultimately led to the differences in both *ProbAct* values (expressed in percentage) and the number of combinations of *ej* against which these molecules were predicted. In the case of the molecules ASP-COLRC-01 and ASP-COLRC-02, they were predicted against a smaller number of CRC cell lines because they present in their structure an aliphatic portion/region that is too large (the tertiary amine moiety plus the 4-methylpiperazine-1-carbonyl fragment). Particularly, this chemical modification unfavorably increases the value of the *D*[*GTI*]*ej* indices *DGT*11 and *DGT*18, being detrimental to the multi-cell anti-CRC activity. Notice that when the 4-methylpiperazine moiety is replaced by the 3-methoxyphenyl fragment, the multi-cell anti-CRC activity increases in molecules from ASP-COLRC-03 to ASP-COLRC-06; i.e., they are predicted against a higher number of CRC cell lines. Yet, as mentioned before, ASP-COLRC-03 is predicted as a multi-cell inhibitor with anti-CRC activity only in 5 out of the 7 combinations of *ej,* while ASP-COLRC-04, ASP-COLRC-05, and ASP-COLRC-06 are predicted against all seven *ej*. The difference between ASP-COLRC-03 and ASP-COLRC-04 is that in the latter, the trifluoromethoxy group has been replaced by the trifluoromethyl group, with the subsequent favorable increase in the value of the *D*[*GTI*]*ej* index *DGT*13. For the case of ASP-COLRC-05 and ASP-COLRC-06, the key is a second methoxy group introduced in the carbon adjacent to the one to which the hydroxyl group is attached; such a chemical modification favorably increases the value of the *D*[*GTI*]*ej* index *DGT*13. It is important to emphasize that *DGT*13 is also the main responsible for the fact that the *ProbAct* values for ASP-COLRC-04 and ASP-COLRC-06 are higher than those related to ASP-COLRC-05 because the trifluoromethyl group is more suitable than the trifluoromethoxy.

The other computational tool, whose predictions are depicted in Table 5, can predict anticancer (GI_50_) against 391 cancer cell lines based on structural information from more than 125,000 chemicals retrieved from ChEMBL and PubChem databases [66,67]. This state-of-the-art computational tool is the web server known as CLC-Pred 2.0 [68]. It is important to emphasize that our PTML-MLP model and CLC-Pred 2.0 are machine learning tools that were created by using different approaches and predict different outcomes. For instance, the PTML-MLP model employs an integrative machine learning approach that, through the use of the *D*[*GTI*]*ej* indices (these are multi-label graph-based indices) as inputs and an MLP network, enables the simultaneous prediction of the anti-CRC activity of any molecule/chemical against seven different CRC cell lines. In contrast, CLC-Pred 2.0 uses as inputs the chemical similarity (fragment-based) descriptors known as the multilevel neighborhood of atoms, with the modeling algorithm being the Naive Bayes classifier [68]; one model has been created to predict activity against each of the seven CRC cell lines. Furthermore, Table 5 illustrated another key difference: the outcome of the prediction; the PTML-MLP model predicts the probability *ProbAct* for a molecule to be active while CLC-Pred 2.0 uses the definitions of probabilities to be active and inactive (*P_a_* and *P_i_*, respectively) based on the similarity of the predicted compound when compared with chemicals in the training set by using the activity cutoff of GI_50_ ≤ 100 nM; therefore, any molecule with *P_a_* > *P_i_* is labeled as active (with predicted activity GI_50_ ≤ 100 nM), and thus, may be considered for future experimental validation.

Because of the difference in approaches used to build the PTML-MLP model and CLC-Pred 2.0, the predictions of multi-cell anti-CRC activity of these two tools should not be expected to fully converge. Nevertheless, because the PTML-MLP model and CLC-Pred 2.0 are classification models based on the same activity endpoint (GI_50_) and assay protocol (sulforhodamine B—SRB, with a time assay of 48 h), a certain agreement in the predictions performed by both tools should be expected in the sense that both tools should be able to predict that the designed molecules exhibit multi-cell anti-CRC activity (against at least 4 out of 7 combinations of *ej*—one per each CRC cell line).

Results from Table 5 show that, when analyzing CLC-Pred 2.0, the six designed molecules were all predicted as active by considering the experimental aspect *ej05*, i.e., against the CRC cell line named KM12 (see Table 3). Particularly, the six designed molecules *P_a_* > *P_i_* for these CRC cell lines, which means that they were predicted by CLC-Pred 2.0 to exhibit GI_50_ ≤ 100 nM. We would like to highlight that the cutoff used by CLC-Pred 2.0 (GI_50_ ≤ 100 nM) is remarkably more rigorous than the one employed to create our PTML-MLP model (GI_50_ ≤ 1900 nM). It is important to highlight that for certain combinations of *ej* (specific CRC cell lines), CLC-Pred 2.0 yielded no prediction results, indicating that there was no chemical similarity information in the machine learning models from CLC-Pred 2.0 to assess the anti-CRC activity of the molecules through the *P_a_* and *P_i_* values. Among the designed molecules, the best predicted is ASP-COLRC-05, while the worst predicted is ASP-COLRC-02; except the latter, all the other molecules were predicted to exhibit multi-cell anti-CRC activity, i.e., a predicted activity value of GI_50_ ≤ 100 nM against at least 4 out of 7 combinations of *ej*. Altogether, the predictions performed by our PTML-MLP model and CLC-Pred 2.0 suggest that the designed molecules may behave as versatile and potent anti-CRC agents.

To assess the chemical novelty of the six designed molecules, we examined prestigious online chemical databases such as ChEMBL [66,69], SureChEMBL [70], eMolecules [71], and ZINC [72,73,74,75]. The purpose here was to check if any of our six designed molecules resembled any of the molecules present in these databases. Thus, for each of the six designed molecules, we performed a similarity search in each of the aforementioned databases, using Tanimoto’s coefficient (*T_c_*). By using the accepted chemical similarity cutoff of *T_c_* > 0.85 [76], we found that there are no molecules in those chemical databases whose structures resemble the ones of our six designed molecules. This demonstrates that our designed molecules have chemical novelty, and, at the same time, pharmacological novelty because no chemical similar to our six designed molecules has been reported to exhibit multi-cell anti-CRC activity.

### 2.4. Druglikeness of the Designed Molecules

Estimating the druglikeness is very important because it can help with the prioritization of those molecules that are more likely to succeed in drug discovery campaigns. One well-established approach to assess the druglikeness is the compliance with certain druglikeness-related rules, such as Lipinski’s rule of five [77] and the Veber guidelines [78]. According to Lipinski’s rule of five, key physicochemical properties are: number of hydrogen bond acceptors and donors (HBA and HBD, respectively), molecular weight (MW), and the logarithm of the octanol-water partition coefficient (logP). According to Lipinski’s rule of five, for a molecule to exhibit drug-like properties, HBA ≤ 10, HBO ≤ 5, MW < 500 Da, and logP ≤ 5. Regarding the Veber guidelines, which criticize the cutoff MW < 500 Da, only the number of rotatable bonds (NRB) and the polar surface area (PSA) are considered; in this sense, a molecule exhibits druglikeness if NRB ≤ 10 and PSA < 140 Å^2^. We employed the AlvaDesc software (version 1.0.22) [79] to calculate all these physicochemical properties (Table 6) to verify the compliance of the six designed molecules with the two aforementioned druglikeness-based rules.

The comparison of the values of the physicochemical properties of the six designed with the cutoff values of the same properties described by Lipinski’s rule of five and Veber guidelines allowed the estimation of the druglikeness of these molecules. Except for ASP-COLRC-05, all the other designed molecules comply with these two druglikeness-related rules. We would like to highlight that although ASP-COLRC-05 violates two aspects of Lipinski’s rule of five, it complies with Veber guidelines. Furthermore, given the current state of modern drug discovery, it is well-established that chemicals presenting two violations of druglikeness-related rules can still be approved as therapeutic drugs by the Food and Drug Administration, can [80]. Thus, altogether, the six designed molecules present druglike properties and can therefore be considered for future synthesis and experimental validation.

## 3. Materials and Methods

### 3.1. Data Retrieval and Curation

Chemical and biological data were retrieved (as a Microsoft Excel-compatible file) from the online repository known as the ChEMBL database [66,69,81,82]. In the extracted data, chemical information for each molecule/chemical was present in the form of a Simplified Molecular Input Line Entry System (SMILES) code; only molecules with molar mass (M) in the range 130–854 g/mol were considered in the present study. In the case of the biological information, this was present as labels of the combinations of *ej* mentioned and discussed in the previous section. Furthermore, the biological activity endpoint (GI_50_) was experimentally determined for each molecule via the SRB assay after 48 h of exposure of the molecules to the CRC cell lines. We eliminated noisy data such as entries that lacked SMILES (or had multi-component SMILES) codes, as well as those where units or values of activity were missing. If a molecule was experimentally tested more than once against the same CRC cell line, all the entries related to that molecule were deleted except the one exhibiting the lowest GI_50_ value. Our dataset contained 5478 cases. Molecules with GI_50_ ≤ 1900 nM were annotated as active, with the observed categorical variable of anti-CRC activity *ACRC*(*ej*) = 1; the other molecules were annotated as inactive, i.e., ACRC(ej) = –1. We would like to emphasize that the activity cutoff of GI_50_ ≤ 1900 nM was chosen because, on one side, it is well-known that chemicals with anticancer activity at least at the low-micromolar range are more likely to exhibit anticancer efficacy later on at the in vivo level [83,84]. This aspect guaranteed a more rigorous search for anti-CRC chemicals. On the other hand, the aforementioned cutoff prevented the excessive imbalance between the number of chemicals/cases annotated as active and the number of those labeled as inactive.

### 3.2. Calculation of the Descriptors

From the SMILES codes of the chemicals (stored in a *.txt file), the software known as MODELAB v1.5 was employed to calculate a series of molecular descriptors known as topological indices [85]. In this sense, three families of topological indices (*TIs*) were calculated. The first family was the weighted bond-based spectral moments [*SM*(*PP*)o], where *PP* was a physicochemical property (based on a bond measure or calculated from atomic contributions) such as bond standard distance (*Std*), bond dipole moment (*Dip*), hydrophobicity (*Hyd*), polar surface area (*Psa*), molar refractivity (*Mol*), Gasteiger-Marsili charges (*Gas*), and atomic weight (*Ato*). Furthermore, the letter “o” was the order (ranging from 1 to 7), i.e., the maximum number of bonds that a fragment/*SG* can have without considering bond order. The second and third families were the Kier-Hall valence connectivity indices [*Xv*(*SG*)m] and the bond-based connectivity indices [*e*(*SG*)m], respectively. In these two families, the subgraph *SG* is a generic fragment that can be further classified into path (*P*), cluster (*C*), path-cluster (*PC*), or a ring (*Ch*). Simultaneously, the order “m” (ranging from 1 to 6) is the exact number of bonds (without considering the bond order) present in a particular *SG* type. In addition to the aforementioned families of *TIs*, a new set of partially normalized *TIs* (*NTIs*) was obtained as the quotient of each *TI* and *L*, with the latter being the number of bonds in a molecule without considering the bond order.

### 3.3. Dataset Splitting and the Box–Jenkins Approach

We then split the 5478 chemicals/cases of the dataset into training and test sets by applying the following procedure. First, we sorted the chemicals according to their increasing GI_50_ values, and then, according to the CRC cells against which they were experimentally tested. Then, the first three chemicals/cases were assigned to the training set while the fourth one was assigned to the test set; we applied that assignment to the entire dataset. We would like to emphasize that the test set was never used for model construction, parameter optimization, threshold selection, or descriptor standardization. Therefore, the molecules/chemicals in the test were completely unknown to the PTML model during training, making the test set a true external hold-out set in the sense of standard machine-learning validation protocols.

Then, we used an adaptation of the Box–Jenkins approach, which allowed us to fuse numeric chemical (structural) information calculated through the different graph-theoretical indices (*GTI*) with the labels provided by the combinations of *ej*:

(1) $$avg[GTI]ej=\frac{1}{n\left(ej\right)}\times \sum_{a=1}^{n\left(ej\right)}{GTI}_{a}\;$$

(2) $$D\left[GTI\right]ej=\left[\frac{GTI-avg\left[GTI\right]ej}{sdv[GTI]}\right]\times \sqrt{p\left(ej\right)}\;$$

It is important to highlight that because *ej* contained the experimental aspects *dt*, *ct*, and *mc*, Equations (1) and (2) were applied to each of them separately. In the case of Equation (1), the symbol “*GTI*” refers to either any *TI* or *NTI*. Notice that the average value, *avg*[*GTI*]*ej* is calculated from *GTI* and *n*(*ej*), with the latter being the number of chemicals/cases in the training set annotated as active, which were experimentally tested by considering the same element of *ej*. Thus, if *n*(*ej*) = *n*(*dt*), then, *avg*[*GTI*]*ej* = *avg*[*GTI*]*dt*. This means that Equation (1) is applied to the element *dt*, and *n*(*dt*) is the active chemicals in the training set tested by considering the same doubling time. The same procedure is applied to the elements *ct* and *mc*. In Equation (2), *sdv*[*GTI*] is the standard deviation calculated from each *GTI* value (only for chemicals in the training set). The term *p*(*ej*) is the a priori probability of finding a chemical tested by considering a defined element of *ej*. In this sense, *p*(*ej*) is the quotient of *n*(*ej*) and the *T (i.e., the* number of training cases mentioned in the previous section). Notice that, as in the case of *n*(*ej*), the *p*(*ej*) values were calculated for *dt*, *ct*, and *mc*, separately.

It is important to highlight that in PTML modeling, the experimental conditions *ej* (*dt*, *ct*, *mc*) do not constitute the perturbations themselves. Rather, they define the condition-specific reference values, i.e., the *avg*[*GTI*]*ej* values calculated in Equation (1). A “perturbation” represents the deviation of an individual descriptor *GTI* from its expected average value, *avg*[*GTI*]*ej*, under that specific experimental condition, as quantified in Equation (2). Thus, the multi-label graph-theoretical index *D*[*GTI*]*ej* is a perturbation descriptor because it encodes the structural change in a molecule relative to the condition-dependent baseline (characterized by *avg*[*GTI*]*ej*).

### 3.4. Development of the PTML Model

We examined the potential discriminatory power of the *D*[*GTI*]*ej* indices. To do so, we employed the computer program known as IMMAN v1.0 [86], which allowed us to calculate for (each *D*[*GTI*]*ej* index) three different metrics from information theory: differential Shannon entropy [87], gain ratio [88], and symmetric uncertainty [89]. Following, IMMAN v1.0 also permitted the calculation of the geometric mean value (GMV) based on the aforementioned metrics. Thus, we ranked the *D*[*GTI*]*ej* indices according to their decreasing GMV; the *D*[*GTI*]*ej* indices with the largest GMV were the ones with the greatest discriminatory power. To reduce information redundancy among the *D*[*GTI*]*ej* indices, we employed the computer program named STATISTICA v13.5.0.17 [90], which allowed us to calculate pair-wise Pearson’s correlation coefficient (*PCC*) values; those *D*[*GTI*]*ej* indices that did not comply with the condition −0.7 < *PCC* < 0.7 were eliminated. Furthermore, given the size of the dataset used in the present study and our previous and vast experience working with PTML models [16,45,91], we arbitrarily concluded that for the development of the PTML-MLP model, we would use as inputs the top 20 non-redundant *D*[*GTI*]*ej* indices, i.e., the *D*[*GTI*]*ej* indices with the highest GMV, which complied with the condition −0.7 < *PCC* < 0.7.

To develop the PTML-MLP model, we used the artificial neural networks (ANN) package of STATISTICA v13.5.0.17, and the key hyperparameters were configured in the following manner. The number of input nodes *I* = 20 (the number of inputted *D*[*GTI*]*ej* indices). Exponential, logistic, and hyperbolic tangent activation functions were evaluated in both hidden and output layers. The minimum and maximum number of neurons in the hidden (*H*) layer were 20 and 60, respectively. The number of output nodes was *O* = 2, i.e., the number of categories (active and inactive) that had to be predicted by the PTML-MLP model. The ANN type of preference was the MLP due to the excellent results obtained by PTML models using this architecture [16,45,91]. As mentioned above, the parameter *ρ* was calculated as a measure of the capacity of the MLP network (PTML-MLP model) to overfit the data [43,92]:

(3) $$\rho =\frac{T}{\left[(I+1)H+(H+1)O\right]}\;$$

Notice that Equation (3) reflects the topology of an MLP network; the parameters *T*, *I*, *H*, and *O* have already been mentioned and explained above.

We trained 1000 MLP networks, retaining 300 of them for further analysis. Among the 300 retained MLP networks, the best of them (PTML-MLP model) was the one exhibiting the highest values of global and local metrics (already explained in the Results and Discussion section) in both training and test sets.

## 4. Conclusions

Among the many neoplastic malignancies, CRC is of great concern due to its elevated mortality and variable degree during prognosis. At the phenotypic level, discovering novel chemicals with multi-cell anti-CRC activity is of paramount importance in the road to finding versatile, more efficacious therapeutics to tackle CRC. Our computational methodology, which combines our PTML-MLP model with the FBTD approach, proposes a chemistry-driven generation of druglike molecules virtually exhibiting multi-cell anti-CRC activity, which holds great promise for future experimental validation through organic synthesis and subsequent determination of the versatility of cytostatic activity against multiple CRC cell lines (multi-cell anti-CRC activity). The present study showcases the applicability of the PTML-FBTD in silico framework in the context of de novo molecular design, opening new horizons for early phenotypic anticancer discovery and beyond.

## Figures and Tables

**Table 1:** Definitions of the different *D*[*GTI*]*ej* indices present in the PTML-MLP model.

Codes ^a,b,c^	Symbology	Definition
*DGT*01	*D*[*NSM*(*Dip*)5]*dt*	Multi-label topological index derived from the normalized bond-based spectral moment of order 5, weighted by the bond dipole moment.
*DGT*02	*D*[*NSM*(*Psa*)1]*dt*	Multi-label topological index derived from the normalized bond-based spectral moment of order 1, weighted by the polar surface area.
*DGT*03	*D*[*NSM*(*Mol*)4]*dt*	Multi-label topological index derived from the normalized bond-based spectral moment of order 4, weighted by the atomic contributions to the molar refractivity.
*DGT*04	*D*[*Ne*(*P*)2]*dt*	Multi-label topological index derived from the normalized bond connectivity of order 2, containing only path subgraphs.
*DGT*05	*D*[*SM*(*Psa*)1]*ct*	Multi-label topological index derived from the bond-based spectral moment of order 1, weighted by the atomic contributions to the polar surface area.
*DGT*06	*D*[*e*(*C*)4]*ct*	Multi-label topological index derived from the bond connectivity of order 4, containing only cluster subgraphs.
*DGT*07	*D*[*SM*(*Hyd*)1]*mc*	Multi-label topological index derived from the bond-based spectral moment of order 1, weighted by the atomic contributions to the hydrophobicity.
*DGT*08	*D*[*SM*(*Psa*)4]*mc*	Multi-label topological index derived from the bond-based spectral moment of order 4, weighted by the atomic contributions to the polar surface area.
*DGT*09	*D*[*SM*(*Mol*)3]*mc*	Multi-label topological index derived from the bond-based spectral moment of order 3, weighted by the atomic contributions to the molar refractivity.
*DGT*10	*D*[*SM*(*Ato*)4]*mc*	Multi-label topological index derived from the bond-based spectral moment of order 4, weighted by the atomic weights.
*DGT*11	*D*[*Xv*(*Ch*)6]*mc*	Multi-label topological index derived from the atom-based valence connectivity of order 6, containing only ring (cycle) subgraphs.
*DGT*12	*D*[*e*(*P*)1]*mc*	Multi-label topological index derived from the bond connectivity of order 1, containing only path subgraphs.
*DGT*13	*D*[*e*(*C*)5]*mc*	Multi-label topological index derived from the bond connectivity of order 5, containing only cluster subgraphs.
*DGT*14	*D*[*e*(*Ch*)5]*mc*	Multi-label topological index derived from the bond connectivity of order 5, containing only ring (cycle) subgraphs.
*DGT*15	*D*[*e*(*Ch*)6]*mc*	Multi-label topological index derived from the bond connectivity of order 6, containing only ring (cycle) subgraphs.
*DGT*16	*D*[*NSM*(*Std*)1]*mc*	Multi-label topological index derived from the normalized bond-based spectral moment of order 1, weighted by the bond standard distance.
*DGT*17	*D*[*NSM*(*Mol*)1]*mc*	Multi-label topological index derived from the normalized bond-based spectral moment of order 1, weighted by the atomic contributions to the molar refractivity.
*DGT*18	*D*[*NXv*(*P*)6]*mc*	Multi-label topological index derived from the normalized atom-based valence connectivity of order 6, containing only path subgraphs.
*DGT*19	*D*[*Ne*(*P*)1]*mc*	Multi-label topological index derived from the normalized bond connectivity of order 1, containing only path subgraphs.
*DGT*20	*D*[*Ne*(*P*)6]*mc*	Multi-label topological index derived from the normalized bond connectivity of order 6, containing only path subgraphs.

^a^ The codes for the *D*[*GTI*]*ej* indices will be used throughout the entire manuscript. ^b^ For the *D*[*GTI*]*ej* indices containing the symbology “*SM*”, the order indicates the maximum number of bonds that a fragment can have (without considering bond order). For the *D*[*GTI*]*ej* indices containing the symbols “*Xv*” and “*e*”, the order is the exact number of bonds (without considering order) present in a fragment. ^c^ The notation *dt* indicates that the *D*[*GTI*]*ej* indices depend on the chemical structure and the cells’ doubling times; likewise, *ct* indicates that the *D*[*GTI*]*ej* indices depend on the chemical structure and the specific CRC cell lines, while *D*[*GTI*]*ej* indices with the notation *mc* depend on the chemical structure and the microsatellite characteristics.

**Table 2:** PTML-MLP model: performance analysis through global metrics.

Symbols ^a^	Training Set	Test Set
*N*_Active_	1846	615
*CC*_Active_	1643	512
*Sn*	89.00%	83.25%
*N*_Inactive_	2262	755
*CC*_Inactive_	2026	635
*Sp*	89.57%	84.11%
*nMCC*	0.892	0.836

^a^ *N*_Active_—Number of chemicals labeled as active; *N*_Inactive_—Number of chemicals labeled as inactive; *CC*_Active_—Number of chemicals correctly classified as active; *CC*_Inactive_—Number of chemicals correctly classified as inactive; *Sn*—Sensitivity (percentage of cases correctly predicted as active); *Sp*—Specificity (percentage of cases correctly predicted as inactive); *nMCC*—Normalized Matthews’ correlation coefficient.

**Table 3:** Biological aspects considered by the PTML-MLP model when predicting anti-CRC activity.

*ej* ^a^	*dt* ^b^	*ct* ^c^	*mc* ^d^
*ej*01	Slow growth	HCC2998	MSS
*ej*02	Fast growth	HT-29	MSS
*ej*03	Fast growth	HCT 15	MSI
*ej*04	Fast growth	HCT 116	MSI
*ej*05	Intermediate growth	KM12	MSI
*ej*06	Intermediate growth	SW620	MSS
*ej*07	Slow growth	COLO 205	MSS

^a^ Codes for the combination of experimental aspects, with each of them considering a defined label belonging to aspect *dt*, one label related to the aspect *ct*, and another label based on the aspect *mc*. ^b^ Labels associated with the different growth rates (doubling times) of each CRC cell line. For the case of the aspect *dt*, the labels were annotated according to the values of doubling times expressed in hours (see the Materials and Methods section). ^c^ Labels for the specific type of CRC cell line. ^d^ Labels involving the microsatellite characteristics of each CRC cell line; the notation “MSS” indicates a stable microsatellite while “MSI” indicates microsatellite instability.

**Table 4:** Relative variability in the values of the different *D*[*GTI*]*ej* indices in the PTML-MLP model.

Codes ^a^	Average Values		Propensity ^b^
	Active	Inactive	
*DGT*01	1.481 × 10^−2^	8.723 × 10^−2^	Decrease
*DGT*02	5.160 × 10^−3^	1.156 × 10^−1^	Decrease
*DGT*03	3.545 × 10^−3^	1.048 × 10^−1^	Decrease
*DGT*04	−2.001 × 10^−2^	1.414 × 10^−1^	Decrease
*DGT*05	2.747 × 10^−3^	4.112 × 10^−2^	Decrease
*DGT*06	6.336 × 10^−3^	2.913 × 10^−2^	Decrease
*DGT*07	−9.067 × 10^−3^	−4.975 × 10^−2^	Increase
*DGT*08	1.135 × 10^−2^	1.840 × 10^−1^	Decrease
*DGT*09	−1.061 × 10^−2^	8.230 × 10^−2^	Decrease
*DGT*10	1.251 × 10^−2^	−2.358 × 10^−2^	Increase
*DGT*11	−7.321 × 10^−3^	−1.911 × 10^−3^	Decrease
*DGT*12	−3.556 × 10^−3^	−1.092 × 10^−1^	Increase
*DGT*13	1.692 × 10^−2^	4.808 × 10^−3^	Increase
*DGT*14	9.767 × 10^−3^	7.377 × 10^−2^	Decrease
*DGT*15	−2.721 × 10^−2^	2.977 × 10^−2^	Decrease
*DGT*16	−1.179 × 10^−2^	−6.458 × 10^−2^	Increase
*DGT*17	−1.816 × 10^−2^	8.865 × 10^−2^	Decrease
*DGT*18	1.347 × 10^−2^	3.415 × 10^−2^	Decrease
*DGT*19	−7.019 × 10^−3^	−6.653 × 10^−2^	Increase
*DGT*20	2.185 × 10^−3^	−1.142 × 10^−1^	Increase

^a^ The codes depicted here are the same as the ones reported in Table 1. ^b^ This refers to the relative variation (decrease or increase) in the value of a defined *D*[*GTI*]*ej* index.

**Table 5:** Predictions of multi-cell anti-CRC activity performed by the PTML-MLP model and CLC-Pred 2.0.

ID ^a^	*ej* ^b^	PTML-MLP Model ^c,d^		CLC-Pred 2.0 (GI_50_ ≤ 100 nM) ^e^	
		*PACRC*(*ej*)	*ProbAct* (%)	*P_a_*	*P_i_*
ASP-COLRC-01	*ej*01	−1	43.43	0.254	0.148
ASP-COLRC-01	*ej*02	1	58.41	0.266	0.124
ASP-COLRC-01	*ej*03	1	55.23	–	–
ASP-COLRC-01	*ej*04	1	66.49	0.209	0.204
ASP-COLRC-01	*ej*05	1	56.13	0.282	0.117
ASP-COLRC-01	*ej*06	−1	42.44	–	–
ASP-COLRC-01	*ej*07	−1	46.02	0.337	0.098
ASP-COLRC-02	*ej*01	1	55.32	–	–
ASP-COLRC-02	*ej*02	1	59.79	–	–
ASP-COLRC-02	*ej*03	−1	39.55	0.224	0.174
ASP-COLRC-02	*ej*04	−1	44.55	–	–
ASP-COLRC-02	*ej*05	−1	39.64	0.254	0.133
ASP-COLRC-02	*ej*06	1	50.60	–	–
ASP-COLRC-02	*ej*07	1	55.38	–	–
ASP-COLRC-03	*ej*01	−1	46.75	0.336	0.097
ASP-COLRC-03	*ej*02	1	59.00	0.302	0.104
ASP-COLRC-03	*ej*03	1	55.76	0.323	0.107
ASP-COLRC-03	*ej*04	1	61.67	0.361	0.097
ASP-COLRC-03	*ej*05	1	55.95	0.369	0.080
ASP-COLRC-03	*ej*06	−1	49.10	0.243	0.153
ASP-COLRC-03	*ej*07	1	50.88	0.353	0.091
ASP-COLRC-04	*ej*01	1	66.31	–	–
ASP-COLRC-04	*ej*02	1	74.59	–	–
ASP-COLRC-04	*ej*03	1	72.67	0.250	0.152
ASP-COLRC-04	*ej*04	1	76.79	0.268	0.148
ASP-COLRC-04	*ej*05	1	73.99	0.204	0.171
ASP-COLRC-04	*ej*06	1	67.95	0.217	0.180
ASP-COLRC-04	*ej*07	1	69.47	–	–
ASP-COLRC-05	*ej*01	1	53.80	0.343	0.093
ASP-COLRC-05	*ej*02	1	61.15	0.302	0.104
ASP-COLRC-05	*ej*03	1	58.83	0.358	0.091
ASP-COLRC-05	*ej*04	1	63.40	0.376	0.090
ASP-COLRC-05	*ej*05	1	58.85	0.397	0.072
ASP-COLRC-05	*ej*06	1	54.71	0.261	0.140
ASP-COLRC-05	*ej*07	1	56.60	0.346	0.094
ASP-COLRC-06	*ej*01	1	76.95	–	–
ASP-COLRC-06	*ej*02	1	79.13	0.189	0.188
ASP-COLRC-06	*ej*03	1	76.56	0.285	0.129
ASP-COLRC-06	*ej*04	1	78.33	0.282	0.138
ASP-COLRC-06	*ej*05	1	77.20	0.228	0.152
ASP-COLRC-06	*ej*06	1	77.28	0.232	0.164
ASP-COLRC-06	*ej*07	1	78.12	–	–

^a^ Codes for the molecules designed as multi-cell anti-CRC agents; these codes coincide with those reported in Figure 5. ^b^ Combinations of experimental aspects as represented in Table 3. ^c^ Predicted categorical activity values obtained by the PTML-MLP model. If *PACRC*(*ej*) = 1 means that the molecule was predicted as active (exhibiting GI50 ≤ 1900 nM); otherwise, the molecule was predicted as inactive, i.e., PACRC(ej) = −1. ^d^ Probability value obtained by the PTML-MLP model for a molecule to be classified as active. ^e^ Probabilities of being active (*P_a_*) and inactive (*P_i_*) by considering the activity cutoff GI_50_ ≤ 100 nM.

**Table 6:** Druglikeness-related physicochemical properties calculated for the six designed molecules.

ID	Physicochemical Properties ^a^							
	MW	HBA	HBD	MlogP	AlogP	AvgLogP	RBN	PSA
ASP-COLRC-01	473.55	10	0	2.304	4.628	3.466	6	61.80
ASP-COLRC-02	435.65	6	0	1.941	3.050	2.496	6	77.87
ASP-COLRC-03	483.48	10	1	2.826	5.393	4.110	7	84.78
ASP-COLRC-04	467.48	9	1	2.773	4.105	3.439	6	75.55
ASP-COLRC-05	513.51	11	1	2.277	5.377	3.827	8	94.01
ASP-COLRC-06	497.51	10	1	2.213	4.089	3.151	7	84.78

^a^ The druglikeness-related physicochemical properties have the following symbols and meanings: MW—molecular weight (expressed in Daltons—Da); HBA—number of hydrogen bond acceptors; HBD—number of hydrogen bond donors; MlogP—the logarithm of octanol-water partition coefficient according to Moriguchi’s method; AlogP—the logarithm of octanol-water partition coefficient according to Ghose–Crippen’s method; AvgLogP—the average value calculated from MlogP and AlogP; RBN—number of rotatable bonds; PSA—polar surface area (expressed in Å^2^).

## Data Availability

Data are provided within the manuscript and Supplementary Information Files.

## Supplementary Materials

The following supporting information can be downloaded at https://www.mdpi.com/article/10.3390/ijms262311453/s1

## References

[1] Sung H., Ferlay J., Siegel R.L., Laversanne M., Soerjomataram I., Jemal A., Bray F. (2021). Global Cancer Statistics 2020: GLOBOCAN Estimates of Incidence and Mortality Worldwide for 36 Cancers in 185 Countries. CA Cancer J. Clin..

[2] Siegel R.L., Kratzer T.B., Giaquinto A.N., Sung H., Jemal A. (2025). Cancer statistics, 2025. CA Cancer J. Clin..

[3] Haynes J., Manogaran P. (2025). Mechanisms and Strategies to Overcome Drug Resistance in Colorectal Cancer. Int. J. Mol. Sci..

[4] Oh J.M., Kim S., Tsung C., Kent E., Jain A., Ruff S.M., Zhang H. (2025). Comprehensive review of the resistance mechanisms of colorectal cancer classified by therapy type. Front. Immunol..

[5] Wang Z., Hulikova A., Swietach P. (2024). Innovating cancer drug discovery with refined phenotypic screens. Trends Pharmacol. Sci..

[6] Jiang C., Yang S., Wang Y., Du L., Niu M.M., Zhang D. (2025). Structure-based design of new potent and highly selective PARP-1 inhibitor for treating colorectal cancer. J. Enzyme Inhib. Med. Chem..

[7] Sharma D., Arumugam S. (2025). A machine learning-Assisted QSAR and integrative computational combined with network pharmacology approach for rational identification of tankyrase inhibitors in colon adenocarcinoma. Comput. Biol. Med..

[8] Pushpaveni C., Hemavathi S., Kurmi S.P.C., Patra B.R., Esther V.A., Yadav C.K., Biradar M.S., Thapa S. (2025). Repurposing terfenadine and domperidone for inhibition of apoptotic gene association in colorectal cancer: A system pharmacology approach integrated with molecular docking, MD simulations, and post-MD simulation analysis. Bioinform. Biol. Insights.

[9] Scalvini L., Tagliazucchi L., Elisi G.M., Zappaterra D., Moschella M.G., Fantini S., Aiello D., Guerrini R., Albanese V., Pacifico S. (2025). Identification of pyrazolo-piperidinone derivatives targeting YAP-TEAD interface 3 as anticancer agents through integrated virtual screening and mass spectrometry proteomics. Eur. J. Med. Chem..

[10] Arooj M., Mateen R.M., Javed M., Ali M., Fareed M.I., Parveen R., Bahadur A., Iqbal S., Mahmood S., Knani S. (2025). Computational screening of phytochemicals targeting mutant KRAS in colorectal cancer. Sci. Rep..

[11] Khalid M., Mateen R.M., Javed M., Ali M., Saqab M.A.N., Parveen R., Asimov A., Bibi S., Bahadur A., Iqbal S. (2025). In-silico analysis of potential phytochemicals targeting mitogen activating protein kinase-14 (MAPK14) gene in colorectal cancer. Sci. Rep..

[12] Oladeji S.M., Conteh D.N., Bello L.A., Adegboyega A.E., Shokunbi O.S. (2025). Rational Design and Optimization of Novel PDE5 Inhibitors for Targeted Colorectal Cancer Therapy: An In Silico Approach. Int. J. Mol. Sci..

[13] Alshahrani M.M. (2025). Structural stability-guided scaffold hopping and computational modeling of tankyrase inhibitors targeting colorectal cancer. PLoS ONE.

[14] Kleandrova V.V., Cordeiro M.N.D.S., Speck-Planche A. (2025). Perturbation-Theory Machine Learning for Multi-Target Drug Discovery in Modern Anticancer Research. Curr. Issues Mol. Biol..

[15] Kleandrova V.V., Cordeiro M.N.D.S., Speck-Planche A. (2023). Optimizing drug discovery using multitasking models for quantitative structure-biological effect relationships: An update of the literature. Expert Opin. Drug Discov..

[16] Kleandrova V.V., Cordeiro M.N.D.S., Speck-Planche A. (2025). In Silico Approach for Antibacterial Discovery: PTML Modeling of Virtual Multi-Strain Inhibitors Against Staphylococcus aureus. Pharmaceuticals.

[17] Velasquez-Lopez Y., Ruiz-Escudero A., Arrasate S., Gonzalez-Diaz H. (2024). Implementation of IFPTML Computational Models in Drug Discovery Against Flaviviridae Family. J. Chem. Inf. Model..

[18] Santiago C., Ortega-Tenezaca B., Barbolla I., Fundora-Ortiz B., Arrasate S., Dea-Ayuela M.A., Gonzalez-Diaz H., Sotomayor N., Lete E. (2022). Prediction of Antileishmanial Compounds: General Model, Preparation, and Evaluation of 2-Acylpyrrole Derivatives. J. Chem. Inf. Model..

[19] Dieguez-Santana K., Casanola-Martin G.M., Torres R., Rasulev B., Green J.R., Gonzalez-Diaz H. (2022). Machine Learning Study of Metabolic Networks vs ChEMBL Data of Antibacterial Compounds. Mol. Pharm..

[20] Vasquez-Dominguez E., Armijos-Jaramillo V.D., Tejera E., Gonzalez-Diaz H. (2019). Multioutput Perturbation-Theory Machine Learning (PTML) Model of ChEMBL Data for Antiretroviral Compounds. Mol. Pharm..

[21] Quevedo-Tumailli V., Ortega-Tenezaca B., Gonzalez-Diaz H. (2021). IFPTML Mapping of Drug Graphs with Protein and Chromosome Structural Networks vs. Pre-Clinical Assay Information for Discovery of Antimalarial Compounds. Int. J. Mol. Sci..

[22] Barbolla I., Hernandez-Suarez L., Quevedo-Tumailli V., Nocedo-Mena D., Arrasate S., Dea-Ayuela M.A., Gonzalez-Diaz H., Sotomayor N., Lete E. (2021). Palladium-mediated synthesis and biological evaluation of C-10b substituted Dihydropyrrolo[1,2-b]isoquinolines as antileishmanial agents. Eur. J. Med. Chem..

[23] Kleandrova V.V., Speck-Planche A. (2020). PTML Modeling for Alzheimer’s Disease: Design and Prediction of Virtual Multi-Target Inhibitors of GSK3B, HDAC1, and HDAC6. Curr. Top. Med. Chem..

[24] Baltasar-Marchueta M., Llona L., M.-Alicante S., Barbolla I., Ibarluzea M.G., Ramis R., Salomon A.M., Fundora B., Araujo A., Muguruza-Montero A. (2024). Identification of Riluzole derivatives as novel calmodulin inhibitors with neuroprotective activity by a joint synthesis, biosensor, and computational guided strategy. Biomed. Pharmacother..

[25] Sampaio-Dias I.E., Rodriguez-Borges J.E., Yanez-Perez V., Arrasate S., Llorente J., Brea J.M., Bediaga H., Vina D., Loza M.I., Caamano O. (2021). Synthesis, Pharmacological, and Biological Evaluation of 2-Furoyl-Based MIF-1 Peptidomimetics and the Development of a General-Purpose Model for Allosteric Modulators (ALLOPTML). ACS Chem. Neurosci..

[26] Diez-Alarcia R., Yanez-Perez V., Muneta-Arrate I., Arrasate S., Lete E., Meana J.J., Gonzalez-Diaz H. (2019). Big Data Challenges Targeting Proteins in GPCR Signaling Pathways; Combining PTML-ChEMBL Models and [(35)S]GTPgammaS Binding Assays. ACS Chem. Neurosci..

[27] Ferreira da Costa J., Silva D., Caamano O., Brea J.M., Loza M.I., Munteanu C.R., Pazos A., Garcia-Mera X., Gonzalez-Diaz H. (2018). Perturbation Theory/Machine Learning Model of ChEMBL Data for Dopamine Targets: Docking, Synthesis, and Assay of New l-Prolyl-l-leucyl-glycinamide Peptidomimetics. ACS Chem. Neurosci..

[28] Tenorio-Borroto E., Castanedo N., Garcia-Mera X., Rivadeneira K., Vazquez Chagoyan J.C., Barbabosa Pliego A., Munteanu C.R., Gonzalez-Diaz H. (2019). Perturbation Theory Machine Learning Modeling of Immunotoxicity for Drugs Targeting Inflammatory Cytokines and Study of the Antimicrobial G1 Using Cytometric Bead Arrays. Chem. Res. Toxicol..

[29] Vazquez-Prieto S., Paniagua E., Solana H., Ubeira F.M., Gonzalez-Diaz H. (2017). A study of the Immune Epitope Database for some fungi species using network topological indices. Mol. Divers..

[30] He S., Segura Abarrategi J., Bediaga H., Arrasate S., Gonzalez-Diaz H. (2024). On the additive artificial intelligence-based discovery of nanoparticle neurodegenerative disease drug delivery systems. Beilstein J. Nanotechnol..

[31] He S., Nader K., Abarrategi J.S., Bediaga H., Nocedo-Mena D., Ascencio E., Casanola-Martin G.M., Castellanos-Rubio I., Insausti M., Rasulev B. (2024). NANO.PTML model for read-across prediction of nanosystems in neurosciences. computational model and experimental case of study. J. Nanobiotechnol..

[32] Ortega-Tenezaca B., Gonzalez-Diaz H. (2021). IFPTML mapping of nanoparticle antibacterial activity vs. pathogen metabolic networks. Nanoscale.

[33] Munteanu C.R., Gutierrez-Asorey P., Blanes-Rodriguez M., Hidalgo-Delgado I., Blanco Liverio M.J., Castineiras Galdo B., Porto-Pazos A.B., Gestal M., Arrasate S., Gonzalez-Diaz H. (2021). Prediction of Anti-Glioblastoma Drug-Decorated Nanoparticle Delivery Systems Using Molecular Descriptors and Machine Learning. Int. J. Mol. Sci..

[34] Dieguez-Santana K., Gonzalez-Diaz H. (2021). Towards machine learning discovery of dual antibacterial drug-nanoparticle systems. Nanoscale.

[35] Urista D.V., Carrue D.B., Otero I., Arrasate S., Quevedo-Tumailli V.F., Gestal M., Gonzalez-Diaz H., Munteanu C.R. (2020). Prediction of Antimalarial Drug-Decorated Nanoparticle Delivery Systems with Random Forest Models. Biology.

[36] Kleandrova V.V., Cordeiro M.N.D.S., Speck-Planche A. (2024). Perturbation Theory Machine Learning Model for Phenotypic Early Antineoplastic Drug Discovery: Design of Virtual Anti-Lung-Cancer Agents. Appl. Sci..

[37] Cabrera-Andrade A., Lopez-Cortes A., Munteanu C.R., Pazos A., Perez-Castillo Y., Tejera E., Arrasate S., Gonzalez-Diaz H. (2020). Perturbation-Theory Machine Learning (PTML) Multilabel Model of the ChEMBL Dataset of Preclinical Assays for Antisarcoma Compounds. ACS Omega.

[38] Cabrera-Andrade A., Lopez-Cortes A., Jaramillo-Koupermann G., Gonzalez-Diaz H., Pazos A., Munteanu C.R., Perez-Castillo Y., Tejera E. (2020). A Multi-Objective Approach for Anti-Osteosarcoma Cancer Agents Discovery Through Drug Repurposing. Pharmaceuticals.

[39] Kleandrova V.V., Cordeiro M., Speck-Planche A. (2025). Perturbation-theory machine learning for mood disorders: Virtual design of dual inhibitors of NET and SERT proteins. BMC Chem..

[40] Tsherniak A., Vazquez F., Montgomery P.G., Weir B.A., Kryukov G., Cowley G.S., Gill S., Harrington W.F., Pantel S., Krill-Burger J.M. (2017). Defining a Cancer Dependency Map. Cell.

[41] Robin T., Capes-Davis A., Bairoch A. (2020). CLASTR: The Cellosaurus STR similarity search tool—A precious help for cell line authentication. Int. J. Cancer.

[42] van der Meer D., Barthorpe S., Yang W., Lightfoot H., Hall C., Gilbert J., Francies H.E., Garnett M.J. (2019). Cell Model Passports-a hub for clinical, genetic and functional datasets of preclinical cancer models. Nucleic Acids Res..

[43] Schneider G., Wrede P. (1998). Artificial neural networks for computer-based molecular design. Prog. Biophys. Mol. Biol..

[44] Chicco D., Jurman G. (2023). The Matthews correlation coefficient (MCC) should replace the ROC AUC as the standard metric for assessing binary classification. BioData Min..

[45] Kleandrova V.V., Cordeiro M., Speck-Planche A. (2025). In Silico Approach for Early Antimalarial Drug Discovery: De Novo Design of Virtual Multi-Strain Antiplasmodial Inhibitors. Microorganisms.

[46] Zhou X., Lin H., Lin H., Shekhar S., Xiong H. (2008). Global Sensitivity Analysis. Encyclopedia of GIS.

[47] Estrada E. (1996). Spectral moments of the edge adjacency matrix in molecular graphs. 1. Definition and applications for the prediction of physical properties of alkanes. J. Chem. Inf. Comput. Sci..

[48] Estrada E. (1997). Spectral moments of the edge adjacency matrix in molecular graphs. 2. Molecules containing heteroatoms and QSAR applications. J. Chem. Inf. Comput. Sci..

[49] Estrada E. (1998). Spectral moments of the edge adjacency matrix in molecular graphs. 3. Molecules containing cycles. J. Chem. Inf. Comput. Sci..

[50] Estrada E. (2008). How the parts organize in the whole? A top-down view of molecular descriptors and properties for QSAR and drug design. Mini Rev. Med. Chem..

[51] Estrada E., Molina E. (2006). Automatic extraction of structural alerts for predicting chromosome aberrations of organic compounds. J. Mol. Graph. Model..

[52] Helguera A.M., Cabrera Perez M.A., Gonzalez M.P., Ruiz R.M., Gonzalez Diaz H. (2005). A topological substructural approach applied to the computational prediction of rodent carcinogenicity. Bioorg. Med. Chem..

[53] Estrada E., Molina E., Perdomo-Lopez I. (2001). Can 3D structural parameters be predicted from 2D (topological) molecular descriptors?. J. Chem. Inf. Comput. Sci..

[54] Estrada E. (2002). Physicochemical Interpretation of Molecular Connectivity Indices. J. Phys. Chem. A.

[55] Kier L.B., Murray W.J., Hall L.H. (1975). Molecular connectivity. 4. Relationships to biological activities. J. Med. Chem..

[56] Kier L.B., Hall L.H. (1976). Molecular connectivity VII: Specific treatment of heteroatoms. J. Pharm. Sci..

[57] Hall L.H., Kier L.B. (1977). Structure-activity studies using valence molecular connectivity. J. Pharm. Sci..

[58] Kier L.B., Hall L.H. (1981). Derivation and significance of valence molecular connectivity. J. Pharm. Sci..

[59] Kier L.B., Hall L.H. (2000). Intermolecular accessibility: The meaning of molecular connectivity. J. Chem. Inf. Comput. Sci..

[60] Kier L.B., Hall L.H. (2001). Molecular connectivity: Intermolecular accessibility and encounter simulation. J. Mol. Graph. Model..

[61] Estrada E. (1995). Edge adjacency relationship and a novel topological index related to molecular volume. J. Chem. Inf. Comput. Sci..

[62] Estrada E. (1995). Edge adjacency relationships in molecular graphs containing heteroatoms: A new topological index related to molar volume. J. Chem. Inf. Comput. Sci..

[63] Estrada E., Rodríguez L. (1999). Edge-Connectivity Indices in QSPR/QSAR Studies. 1. Comparison to Other Topological Indices in QSPR Studies. J. Chem. Inf. Comput. Sci..

[64] Estrada E. (1999). Edge-Connectivity Indices in QSPR/QSAR Studies. 2. Accounting for Long-Range Bond Contributions. J. Chem. Inf. Comput. Sci..

[65] Estrada E., Guevara N., Gutman I. (1998). Extension of Edge Connectivity Index. Relationships to Line Graph Indices and QSPR Applications. J. Chem. Inf. Comput. Sci..

[66] Zdrazil B., Felix E., Hunter F., Manners E.J., Blackshaw J., Corbett S., de Veij M., Ioannidis H., Lopez D.M., Mosquera J.F. (2024). The ChEMBL Database in 2023: A drug discovery platform spanning multiple bioactivity data types and time periods. Nucleic Acids Res..

[67] Kim S., Chen J., Cheng T., Gindulyte A., He J., He S., Li Q., Shoemaker B.A., Thiessen P.A., Yu B. (2019). PubChem 2019 update: Improved access to chemical data. Nucleic Acids Res..

[68] Lagunin A.A., Rudik A.V., Pogodin P.V., Savosina P.I., Tarasova O.A., Dmitriev A.V., Ivanov S.M., Biziukova N.Y., Druzhilovskiy D.S., Filimonov D.A. (2023). CLC-Pred 2.0: A Freely Available Web Application for In Silico Prediction of Human Cell Line Cytotoxicity and Molecular Mechanisms of Action for Druglike Compounds. Int. J. Mol. Sci..

[69] Mendez D., Gaulton A., Bento A.P., Chambers J., De Veij M., Felix E., Magarinos M.P., Mosquera J.F., Mutowo P., Nowotka M. (2019). ChEMBL: Towards direct deposition of bioassay data. Nucleic Acids Res..

[70] Papadatos G., Davies M., Dedman N., Chambers J., Gaulton A., Siddle J., Koks R., Irvine S.A., Pettersson J., Goncharoff N. (2016). SureChEMBL: A large-scale, chemically annotated patent document database. Nucleic Acids Res..

[71] Gubernator K., James C.A., Gubernator N. eMolecules. California, USA. https://www.emolecules.com/.

[72] Irwin J.J., Tang K.G., Young J., Dandarchuluun C., Wong B.R., Khurelbaatar M., Moroz Y.S., Mayfield J., Sayle R.A. (2020). ZINC20-A Free Ultralarge-Scale Chemical Database for Ligand Discovery. J. Chem. Inf. Model..

[73] Sterling T., Irwin J.J. (2015). ZINC 15--Ligand Discovery for Everyone. J. Chem. Inf. Model..

[74] Irwin J.J., Sterling T., Mysinger M.M., Bolstad E.S., Coleman R.G. (2012). ZINC: A free tool to discover chemistry for biology. J. Chem. Inf. Model..

[75] Irwin J.J., Shoichet B.K. (2005). ZINC– a free database of commercially available compounds for virtual screening. J. Chem. Inf. Model..

[76] Maggiora G., Vogt M., Stumpfe D., Bajorath J. (2014). Molecular similarity in medicinal chemistry. J. Med. Chem..

[77] Lipinski C.A., Lombardo F., Dominy B.W., Feeney P.J. (2001). Experimental and computational approaches to estimate solubility and permeability in drug discovery and development settings. Adv. Drug Deliv. Rev..

[78] Veber D.F., Johnson S.R., Cheng H.Y., Smith B.R., Ward K.W., Kopple K.D. (2002). Molecular properties that influence the oral bioavailability of drug candidates. J. Med. Chem..

[79] Mauri A., Roy K. (2020). alvaDesc: A Tool to Calculate and Analyze Molecular Descriptors and Fingerprints. Ecotoxicological QSARs.

[80] Pathania S., Singh P.K. (2021). Analyzing FDA-approved drugs for compliance of pharmacokinetic principles: Should there be a critical screening parameter in drug designing protocols?. Expert Opin. Drug Metab. Toxicol..

[81] Gaulton A., Bellis L.J., Bento A.P., Chambers J., Davies M., Hersey A., Light Y., McGlinchey S., Michalovich D., Al-Lazikani B. (2012). ChEMBL: A large-scale bioactivity database for drug discovery. Nucleic Acids Res..

[82] Mok N.Y., Brenk R. (2011). Mining the ChEMBL database: An efficient chemoinformatics workflow for assembling an ion channel-focused screening library. J. Chem. Inf. Model..

[83] Holbeck S.L., Collins J.M., Doroshow J.H. (2010). Analysis of Food and Drug Administration-approved anticancer agents in the NCI60 panel of human tumor cell lines. Mol. Cancer Ther..

[84] Johnson J.I., Decker S., Zaharevitz D., Rubinstein L.V., Venditti J.M., Schepartz S., Kalyandrug S., Christian M., Arbuck S., Hollingshead M. (2001). Relationships between drug activity in NCI preclinical in vitro and in vivo models and early clinical trials. Br. J. Cancer.

[85] Estrada E., Gutiérrez Y. (2004). MODESLAB, v1.5; Santiago de Compostela, Spain. https://insilicomoleculardesign.com/modeslab/.

[86] Urias R.W., Barigye S.J., Marrero-Ponce Y., Garcia-Jacas C.R., Valdes-Martini J.R., Perez-Gimenez F. (2015). IMMAN: Free software for information theory-based chemometric analysis. Mol. Divers..

[87] Stahura F.L., Godden J.W., Bajorath J. (2002). Differential Shannon entropy analysis identifies molecular property descriptors that predict aqueous solubility of synthetic compounds with high accuracy in binary QSAR calculations. J. Chem. Inf. Comput. Sci..

[88] Quinlan J.R. (1986). Induction of decision trees. Mach. Learn..

[89] Press W.H., Flannery B.P., Teukolsky S.A., Vetterling W.T. (1988). Numerical Recipes in C: The Art of Scientific Computing.

[90] TIBCO-Software-Inc (2018). STATISTICA (Data Analysis Software System).

[91] Kleandrova V.V., Cordeiro M.N.D.S., Speck-Planche A. (2025). Perturbation-Theory Machine Learning for Multi-Objective Antibacterial Discovery: Current Status and Future Perspectives. Appl. Sci..

[92] Manallack D.T., Livingstone D.J., A.-Razzak M., Glen R.C., van de Waterbeemd H. (1994). Neural Networks and Expert Systems in Molecular Design. Advanced Computer-Assisted Techniques in Drug Discovery.

